# Inaugural experience and early results of minimally invasive approach in cardiac surgery in Auvergne region, France

**DOI:** 10.34172/jcvtr.2020.15

**Published:** 2020-05-22

**Authors:** Adama Sawadogo, Hoang Nam Nguyen, Nicolas D'Ostrevy, Lionel Camilleri, Kasra Azarnoush

**Affiliations:** ^1^Department of Cardiovascular Surgery, University Hospital of Clermont-Ferrand 63003 Clermont-Ferrand, France; ^2^Department of Surgery, University Hospital of Tengandogo, Burkina Faso

**Keywords:** Minimally Invasive Surgery, Cardiac Surgery, Valve Surgery, Cor Knot, Auvergne

## Abstract

***Introduction:*** Minimally invasive approach in cardiac surgery has become an established and common technique in many cardiac surgery centres throughout the world. We report how we safely introduced minimally invasive approach in cardiac surgery in our department and we aim to demonstrate that this approach is feasible in any medium-size cardiac surgical centre.

***Methods:*** it consisted of retrospective and descriptive study on 60 patients who underwent minimally invasive mitral valve (45) or aortic valve surgery (15) from January 2017 to Februry 2018. The approach was 3 to 6-cm right thoracotomy through the 4th and 5th intercostal space. The Cor-KnotTM system was used to tie the knots of the prosthesis in case of mitral valve replacement and aortic valve replacement and the ring if mitral valve repair.

***Results:*** There was no conversion of thoracotomy to sternotomy. The average duration in ICU was 4.3± 2.3 days and 3.3 ± 1.5 respectively for mitral and aortic valve surgery. Four mitral patients and 1 aortic patient were reoperated for bleeding. No in-hospital death was observed. The postoperative discharge echocardiogram was normal in 95.6% of the mitral valve patients the trans-aortic mean gradient for the aortic valve patients was 16.3 ± 6 mm Hg. The thirty-day mortality was zero. In the majority of the patients, the scar of the thoracotomy were almost unseen.

***Conclusion:*** It is possible to safely implement this new approach in any mid-size cardiac centers. The use of modern technology such as 3D video and Cor Knot allows achievement of excellent short term outcomes.

## Introduction

Hugo Vanermen (Aalst, Belgium) is the most known pioneer of the minimally invasive approach in cardiac surgery as he defined and described the basics of this technique.^1,2^ The other major contributor to this approach is Jean Francois Obadia (Lyon, France) who adopted transthoracic aortic clamping rather than of intra-aortic balloon of Vanermen.^3^ Another great contributor to minimally invasive approach for aortic valve surgery was Mattia Glauber (Milan, Italy) who described a right straight incision with a mean length of 6–8 cm, through the third intercostal space, which gives a perfect valve exposure and working field to replace the aortic valve without any particular difficulties. ^4^ One constraint of minimally invasive surgery is the need for remote knot tying, which is typically accomplished with the use of a knot pusher. This method is usually difficult to do with large variations depending upon surgeons’ experience. The COR-KNOT technology (LSI SOLUTIONS, Victor, NY USA) was developed to address this unmet need in the field of minimally invasive valve surgery. This device has been widely accepted by cardiac surgeons for the recognized benefits of time savings, simplicity, and reliability.^5^ In Auvergne region, France, traditional approach for valve surgery by sternotomy has been performed since 1985.^6^ The minimally invasive technique started in January 2017 with 3D video assisted approach with Cor Knot for mitral and tricuspid valve surgery. A year later, we started minithoracotomy approach for aortic valve surgery. The aims of this work are, firstly to report how we safely introduced minimally invasive approach in cardiac surgery in our department which is the only cardiac centre of the region. Secondly, we aim to demonstrate that this approach is possible anywhere in the world in a medium-size cardiac surgical centre. Lastly, we would show that depending on the curve of learning and modern devices (3D video, Cor-Knot), it is possible to achieve good outcomes.

## Materials and Methods

### Team training

The project of video assisted mitral valve (MV) surgery in our department started in 2015. A senior cardiac surgeon, 2 anesthetists, a team leader, 4 scrub nurses and two perfusionists were designed to get trained as a team. Then, they went several times to other cardiac centers in France and Germany where this approach has been performing for short term training. On January 2017, the team started to perform video assisted mitral surgery. Later on December 2017, the first aortic replacement was performed through a minithoracotomy.

### Population

From January 2017 to February 2018, 45 patients consecutively underwent minimally invasive mitral surgery in our department. Following these patients, 15 others were operated for aortic valve replacement (AVR)through a minithoracotomy. All the patients were living in France and 2 were under hemodialysis for chronic kidney disease.

### Surgical procedure

#### 3D video assisted mitral surgery

**Figure 1:**
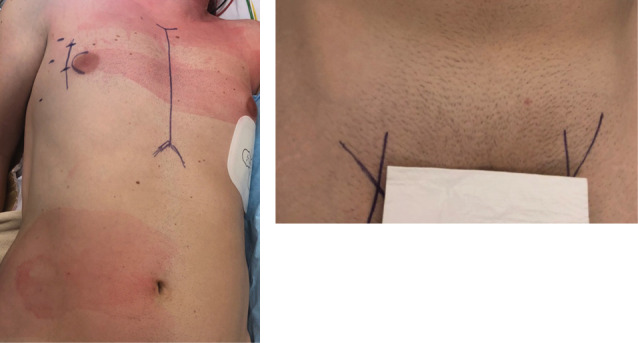
Skin marking.

**Figure 2:**
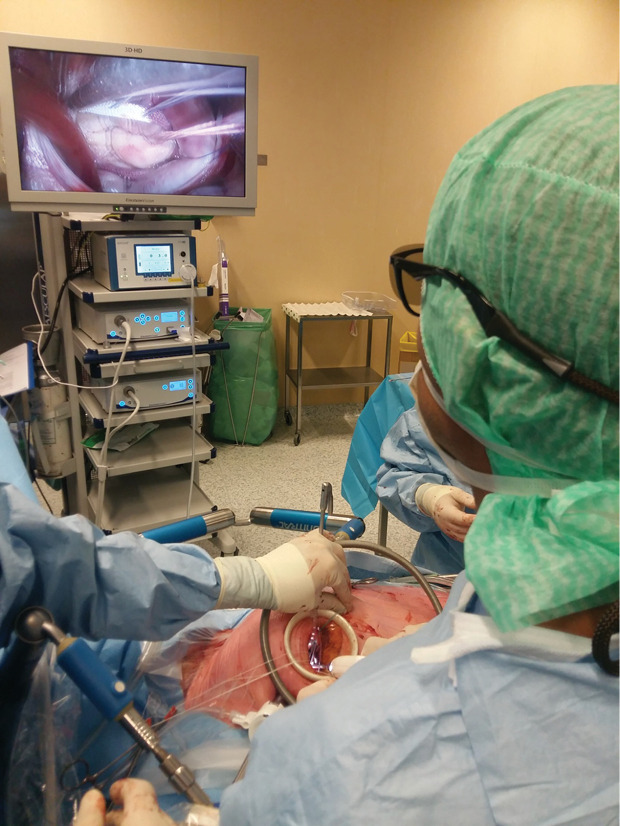
3D HD video assisted mitral valve repair.

**Figure 3:**
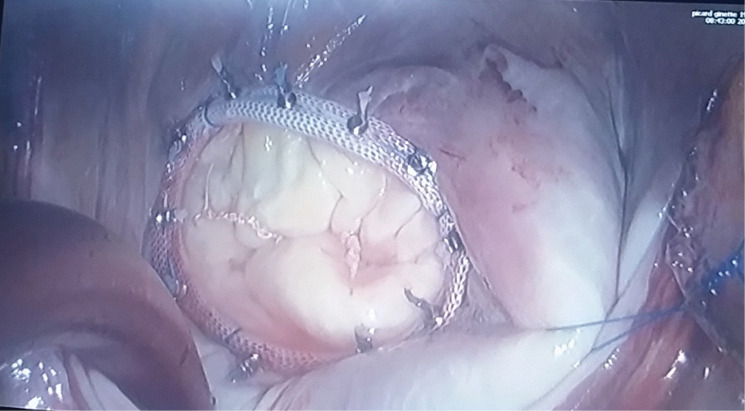
Mitral valve repair showing sutures tied and cut by Cor-Knot.

The same approach was used for all the patients: supine position with a small roller that is longitudinally placed under the right side of the trunk. Transesophageal echocardiography is the gold standard. Defibrillation pads are placed in lateral and posterior position. Surgical landmarks at the groin and thorax are indicated by skin marker (Figure 1). The skin incision is firstly performed at the right groin on 3 cm, dissection until the femoral vessels. Then 4-6 cm incision under the inferior mammary groove through the right 4^th^ intercostal space. Echoguided cannulation of the femoral vein (generally 25 Fr cannula), then the femoral artery with 19 Fr cannula. In case of concomitant tricuspid valve repair, a second venous cannula is introduced into the right jugular vein. A 10-mm port is introduced through the 4^th^ intercostal space (IS) on the anterior axillary line for camera 3D HD Einstein Vision^®^. Then a 5-mm port is placed on the 5^th^ IS for venting. Once the cardiopulmonary bypass (CPB) is started, ventilation is stopped, the pericardium is opened and pulled by 4 pericardial traction sutures, placement of cardioplegia and aortic root sucker cannula on the ascending aortic with double purge string. The ascending aorta is directly clamped using a Chitwood or intrathoracic clamping of the aorta (Glauber – Aortic- Clamp). We use Custodiol cardioplegia (EUSA Pharma, Limonest, France): 1 shot, anterograde in the ascending aorta. Mild hypothermia (32°C) is used during cross-clamping. Carbon dioxide (CO_2_) is started before left atriotomy and insertion of the vent through. The left atrium is entered along the Sondergaard groove. MV is retracted by ValveGate^TM^ retractor (Figure 2). The valve procedure is then performed using standard technique. Cor- Knot^7^ system is used in both MVR and or MV repair to fasten the knot tying (Figure 3). The pacemaker electrodes must be placed on the inferior side of the right ventricle before cross-clamp removal. We prevent air embolization by using carbon dioxide (0.5 l/min) and a careful deairing technique. The transesophageal echocardiography helps by assessing the air condition in the left ventricle and post repair valve mitral. The pericardium is closed fully, leaving in two chest drains, 1 in the pericardium and the other in the right pleural space.

#### Aortic valve surgery by mini thoracotomy

Patients’ positioning, skin marking at the groins are the same as described previously. The skin incision is performed on the 2nd or 3rd right IS. The right internal mammary artery and vein are sacrificed systematically. The ribs are retracted always by Cor-Valve retractors (Coroneo, Montreal, Quebec, Canada). Exposure of pericardium, CPB, vent placement and CO_2_ are conducted in the same way like in mitral surgery. Aortic clamping is done by Chitwood clamp. The ascending aorta is opened by a J-type incision. AVR is performed using the standard technique with the use of U-shaped pledgeted sutures. Cor Knot system is also used to tie the knots. The rest of the procedure runs as usual.

### Management of postoperative pain

Minimally invasive cardiac surgery is an expanding and evolving specialty within cardiac surgery with purported benefits of fewer transfusions, shorter length of stay, and faster recovery. Although these advantages are attributed largely to surgical technique, the anesthesiologist can play a large role in other aspects of management in these cases, specifically postoperative pain control.^8^ The neuroaxial techniques such the paravertebral block (PVB),^9^ erector spinae plane (ESP)^10^ block and pectoral fascial blocks (PECS)^11^ seems to be safe and prometheus techniques for intra- and postoperative pain management. PVB or ESP block the techniques used in our institution with good results. The blocks are ultrasound guided and performed with ropivacaine 5 mg/mL-20 mL single shoot injection at T4-T5 right paravertebral region. The IV Dexamethasone 8mg is used to prolong the efficacity of the neuroaxial block. Postoperative analgesia is completed with paracetamol, anti-inflammatory non-steroid drugs and opioids if needed.

### Statistical analysis

Statistical analysis was performed using Stata software (version 13; StataCorp, College Station, Texas, USA). Categorical parameters were expressed as frequencies and associated percentages and continuous data as mean ± standard deviation or as median [interquartile range], according to statistical distribution. The Gaussian distribution was verified by the Shapiro-Wilk test.

## Results

### Preoperative and operative characteristics

The preoperative data of all included patients are summarized in Table 1. By the time passing, the number of patients who underwent minimally invasive cardiac surgery has increased especially during the last months of the study due to encouraging early outcomes and the progressive mastering of the technique. Two patients (one mitral and one aortic patient) who were initially included for minimally invasive approach finally underwent sternotomy due to lung adherences to thoracic wall. All the cases were electively performed by one senior surgeon. Patients included for minithoracotomy AVR were associated with more cardiovascular risk factors. There was no approach conversion and operative mortality was nil. Table 2 shows the core of intraoperative parameters of all the patients. Relating to mitral regurgitation, there were no rheumatic or infectious etiologies.

**Table 1:** Preoperative charcteristics

**Characteristic**	**Mitral valve**	**Aortic valve**
Number	45	15
Age (years), mean ± SD	61±13.1	62.2±14.1
Men, n (%)	30 (66.7)	11 (73.3)
BMI (kg/m^2^), mean ± SD	23.36 ±3.4	24.2 ± 3.86
Diabetes, n (%)	0 (0.0)	4 (26.7)
Smoker, n (%)	4 (8.9)	4 (26.7)
Dyslipidemia, n (%)	9 (20)	8 (53.4)
Respiratory disease, n (%)	2 (4.4)	0 (0.0)
Pulmonary Hypertension, n (%)		
Normal	23 (51.1)	14 (93.3)
Moderate	17 (37.8)	1 (6.7)
Severe	5 (11.1)	0 (0.0)
Renal function		
Creatinine (µmol/L), mean ± SD	86 ± 20	81.4 ± 17.8
Creatinine clearance (ml/min), mean ± SD	78.8 ± 29	83.9 ± 26.4
Euroscore 2, mean ± SD	1.5 ± 1.4	0.9 ± 0.3
Class of NYHA, n (%)		
1	4 (8.9)	5 (33.3)
2	29 (64.4)	10 (66.7)
3	11 (24.4)	0 (0.0)
4	1 (2.3)	0 (0.0)
Heart rhythm, n (%)		
Sinus rhythm	38 (81.5)	15 (0.0)
Atrial fibrillation	6 (16.3)	0 (0.0)
Pacemaker	1 (2.2)	0 (0.0)
Preoperative echocardiogram		
Dilated left atrium, n (%)	44 (97.7)	-
LV Ejection fraction (%), mean ± SD (%), LV diastolic diameter (mm), mean ± SD	62.3 ± 8.1	63.9 ± 11.3
LV systolic diameter (mm), mean ± SD	49.5 ± 23.7	27.27 ± 9.2
Grade of mitral regurgitation, n (%)		
0	1 (2.3)	9 (60)
1	0 (0.0)	6 (40)
2	2 (4.4)	0 (0.0)
3	4 (8.9)	0 (0.0)
4	38 (84.4)	0 (0.0)
Grade of tricuspid regurgitation		
0	18 (42.9)	
1	19 (45.2)	
2	3 (7.1)	
3	1 (2.4)	
4	1 (2.4)	
PASP (mm Hg), mean ± SD	37.6 ± 13.7	30.4 ± 6.9
Grade of aortic regurgitation, n (%)		
0	-	7 (46.7)
1		4 (26.7)
2		1 (6.6)
3		1 (6.6)
4		2 (13.4)
Aortic stenosis, n (%)		13 (86.7)
Trans-aortic mean gradient (mm Hg), mean ± SD	-	38.5 ± 10.7
Aortic EOA (cm^2^)	-	0.7 ± 0.1

**Table 2:** Intraoperative data

	**Mitral valve**	**Aortic valve**
Etiology of valve disease, n (%)		
Degenerative	44 (97.8)	14 (93.3)
Fibroelastoma	1 (2.2)	-
Leaflet fenestration congenital	-	1 (6.7)
Aortic valve morphology, n (%)		
Bicuspid		4 (26.7)
Tricuspid	-	11 (73.3)
Mitral valve procedure, n (%)		
MV repair	28	
Ring repair	4 (8.9)	
Leaflet resection	20 (44.4	
Neochordae	3 (6.7)	
Fibroelastoma resection	1 (2.2)	
MV replacement	17	
Bioprosthesis	13 (28.8)	
Mechanical	4 (8.9)	
Associated tricuspid valve repair	3 (6.7)	-
Type of prosthesis used for AVR n (%)		
Bioprosthesis		12 (80)
Mechanical	-	3 (20)
CPB time (min), median [IQR]	210 [190; 233]	151[129; 175]
Cross clamping time (min), median [IQR]	123 [104; 139]	96 [78; 115]

### In-hospital outcomes, discharge echocardiogram and 30-day outcomes

Postoperative outcomes and discharge echocardiogram are summarized in Table 3. No postoperative infection was reported. The vast majority of the patients did not present cardiac symptoms on their discharge from hospital. Discharge echocardiogram showed preserved left ventricular (LV) function. Result for MV repair was excellent in 96% and there was no aortic leakage following AVR. The thirty-day mortality was zero.

**Table 3:** Postoperative outcomes and discharge echocardiogram

	**Mitral valve** **(n = 45 )**	**Aortic valve** **( n = 15)**
ICU stay (days), mean ± SD	4.3± 2.3	3.3 ± 1.5
Hospital stay (days), mean ± SD	9.8 ± 3.5	9.9 ± 3.5
In-hospital morbidity		
Reoperation for bleeding, n (%)	4 (8.9)	1 (6.7)
Pacemaker placement, n (%)	3 (6.7)	0 (0.0)
Occlusion of circumflex, n (%)	1 (2.2)	0 (0.0)
Pericardial effusion, n (%)	1 (2.2)	0 (0.0)
Discharge echocardiogram		
LV Ejection fraction (%), mean ± SD	55.7 ± 7.2	59.6 ± 13.4
Grade of mitral regurgitation, n (%)		
[0 - 2]	43 (95.6)	
[3 - 4]	2 (4.4)	
Trans mitral mean gradient (mm Hg), mean ± SD	4.9 ± 2	-
Grade of aortic regurgitation, n (%)		
[0 - 2]	43 (95.6)	15 (100)
[3 - 4]	2 (4.4)	0 (0.0)
Trans-aortic mean gradient (mm Hg), mean ± SD		16.3 ± 6

### Short-term follow-up

**Figure 4:**
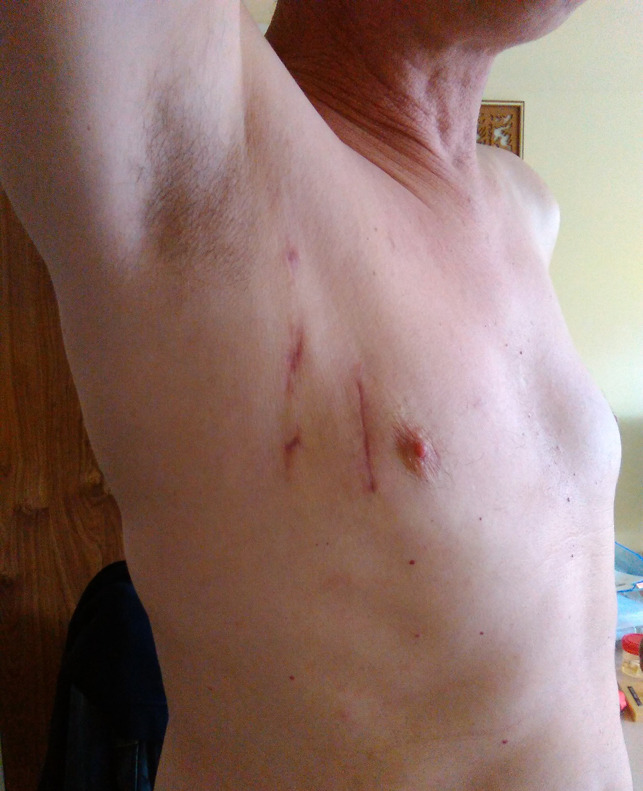
Post-operative scar.

The mean follow-up, all patients included was 3 months ± 1.9. No case of mortality was reported during this period. Many patients declared to be “satisfied” for their operation as they had good quality of life. In the majority of the patients, the scar of the thoracotomy were almost unseen (Figure 4).

## Discussion

The inaugural experience of minimally invasive approach in cardiac surgery in Clermont-Ferrand is encouraging. The key target of this study is to show how we used this new approach to get safe results for our patients. We humbly recognized that the series is small in term of study population and short in follow-up but it seemed for us that the experience deserves being shared.

Hospital mortality is lower than standard valve surgery in our department as reported in a previous study by Camilleri and colleagues (2.2%) for mechanical MVR.^12^ Recently, another study by Mujtabaet al^13^ reported 2.4% of mortality in 624 patients who underwent sternotomy AVR. Our CPB and cross-clamp times are longer than the majority of large series. For instance, Muneretto et al^14^ who reported their experience in video-assisted MV repair in advanced Barlow disease have found 98 minutes ± 23 and 131 minutes ± 41 minutes, respectively for cross-clamp and CPB times. Indeed, procedure time will shorten with the curve of learning. To illustrate, our results are similar to the findings in the early series of Vanermen in which mean cross-clamp and perfusion times were 103 minutes (range: 24-160 minutes) and 140 minutes (range: 75-215 minutes) for minimally invasive AVR.^15^ In our department, we adopted the systematic use of Cor Knot that aims to reduce operative time. We did not experience any complication relating to femoral vessels cannulation. But in the study of Pozzi et al, complications on the arterial side were 1.6% of bleeding, 0.6% retroperitoneal bleeds during CPB, 2% bleeding episodes after removal of the arterial cannula, 0.3% arteriovenous fistula, and 0.3% patient had a transitory claudication due to a superficial femoral artery thrombosis progressively compensated by the collateral circulation.^16^

For a starting experience and in comparison with other series of minimally invasive approach, the overall morbidity rate in our study is acceptable in term of bleeding, infection, cardiac and cerebrovascular events. The discharge echocardiogram findings also corroborate most of studies that reported good results of MV surgery or AVR.^14,15^ In comparison with conventional valve surgery in our department^12^, hospital stay was lower after minimally invasive surgery. The follow-up was relatively short but in general, valves remained stable over the few months as no significant difference was noticed in relation to symptoms, complications, echocardiographic findings or mortality. These days, the esthetic benefits of this approach in addition to clinical safety have made it the approach of choice for valve surgery in our department.

## Conclusion

It is possible to safely implement this new approach in any mid-size cardiac centers. The use of modern technology such as 3D video and Cor Knot allows achievement of excellent short term outcomes.

## Conflicts of Interest

The authors have no conflicts of interest to declare.

## Ethical approval

The study was approved by institutional ethics committee of the University Hospital of Clermont-Ferrand (ETSH1200330A) and informed consent was taken from all the patients.

## Funding

This research received no specific grant from any funding agency in the public, commercial or not-for-profit sectors.
